# Supplementary material for: Impact of β-defensin 103 (DEFB103) copy number variation on bull sperm parameters and post-insemination uterine gene expression
Source: PLoS One. 2025 Feb 25;20(2):e0319281. doi: 10.1371/journal.pone.0319281 (PMC11856272; doi:10.1371/journal.pone.0319281)

Supplementary figure S1 The Principal Component Analysis (PCA) plot showing the distribution of RNA-seq samples, where colours and shape indicates the three different genotypes, namely, high CN (5 heifers), intermediate CN (6 heifers) and low CN (7 heifers).

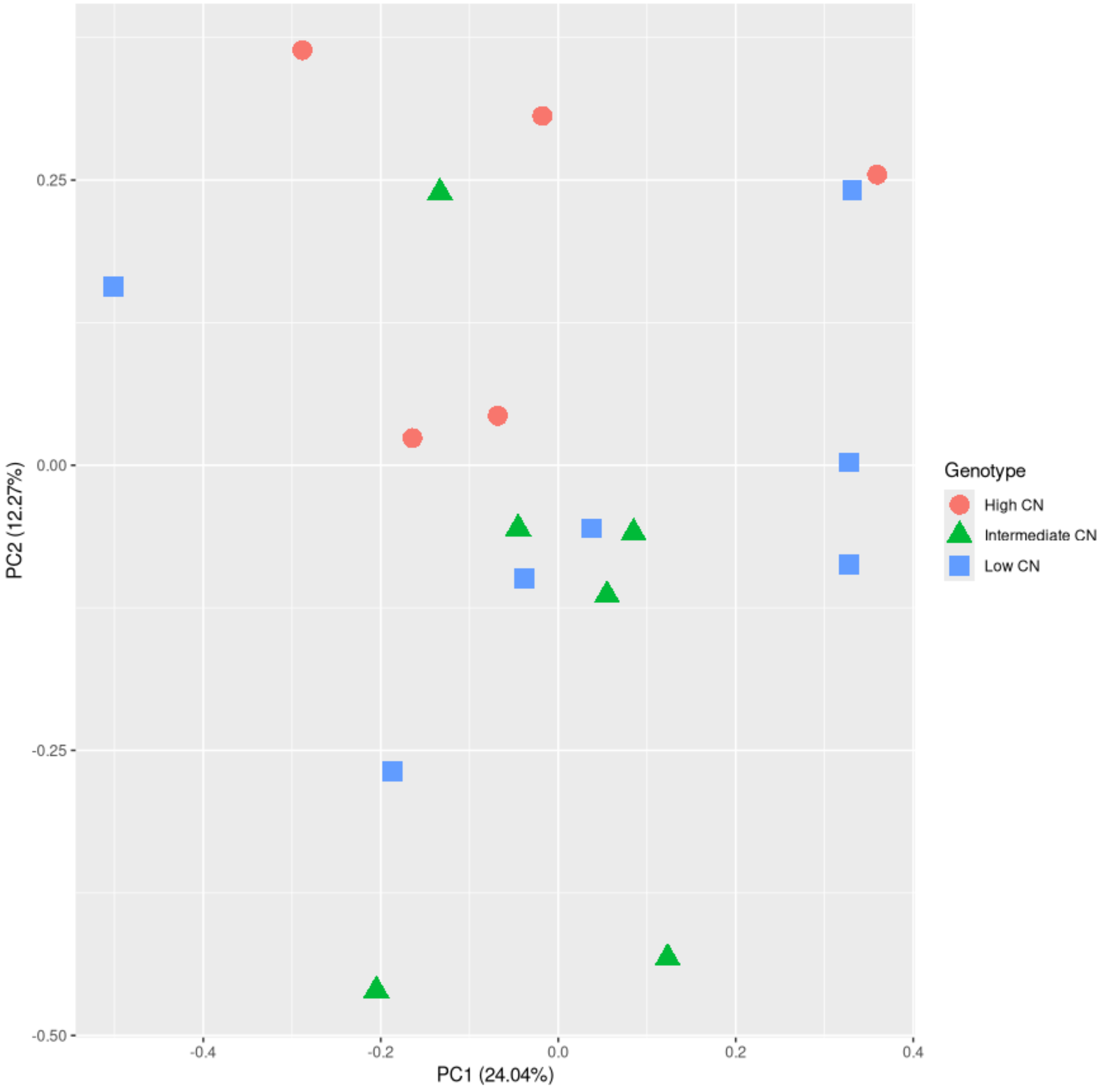

Supplement: S1 Fig — (PDF) [file pone.0319281.s001.pdf]
