# Supplementary material for: Impact of β-defensin 103 (DEFB103) copy number variation on bull sperm parameters and post-insemination uterine gene expression
Source: PLoS One. 2025 Feb 25;20(2):e0319281. doi: 10.1371/journal.pone.0319281 (PMC11856272; doi:10.1371/journal.pone.0319281)
Supplement: Attachment — Submitted filename: DEFB103 paper_Final-To submit_Nov 12 KM (reviewed).docx [file pone.0319281.s004.docx]

**Impact of β-defensin 103 (*DEFB103*) copy number variation on bull sperm parameters and post-insemination uterine gene expression**

Ozge Sidekli ^1^, Edward J. Hollox^1^, Sean Fair^2^ and Kieran G. Meade^3,4,5^

^1^ Department of Genetics, Genomics and Cancer Sciences, University of Leicester, Leicester, United Kingdom

^2^ Laboratory of Animal Reproduction, Department of Biological Sciences, Bernal Institute, Faculty of Science and Engineering, University of Limerick, Limerick, Ireland

^3^ School of Agriculture and Food Science, University College Dublin, Ireland

^4^ UCD Conway Institute of Biomolecular and Biomedical Research, University College Dublin, Belfield, D04 V1W8, Dublin, Ireland

^5^ UCD Institute of Food and Health, University College Dublin, Belfield, C15 PW93, Dublin, Ireland.

**Correspondence:** [kieran.meade@ucd.ie](mailto:kieran.meade@ucd.ie)

**Abstract**

Pregnancy rates for elite bulls used in artificial insemination (AI) can vary significantly and therefore the identification of molecular markers for fertility and targets to improve bull selection is important. β-defensin peptides have diverse regulatory roles in sperm function across multiple species. In this study, Holstein-Friesian bulls were screened based on field fertility data to identify two groups (High and Low fertility (HF and LF, respectively)) of n=10 bulls per group which were genotyped for copy number variation (CNV) in the *DEFB103* gene. Overall, low *DEFB103* copy number (CN) was associated with increased sperm motility across all bulls (n=20, p<0.05). As genetic diversity of *DEFB103* CN was only apparent in the LF group, three bulls per CNV class (low, intermediate and high CN) were chosen for more detailed comparative functional analysis. Low CN exhibited higher binding to the oviductal epithelium, while high CN increased sperm membrane fluidity *in vitro* (p<0.05). To investigate the functional effect of *DEFB103* CNV on the uterine response *in vivo*, 18 heifers were inseminated with sperm from bulls with low, intermediate and high CN. Transcriptomic analysis on uterine tissue harvested 12 h post-insemination showed differential expression of 58 genes (FDR<0.1) involved in sperm migration, immune signalling and chemotaxis. Although field fertility results from a complex number of interactive factors, these novel results confirm a role for *DEFB103* CN in both sperm function and the uterine response to bull sperm, thereby potentially contributing to pregnancy outcomes in cattle.

**Introduction**

Fertility is critically important for the livestock industry, as it directly affects production outcomes as well as underpinning the economic sustainability of farming systems [1]. This is particularly true within a seasonal calving system, where a cow must produce a calf per year and calve early in early spring to maximise the amount of milk produced from low-cost grass forage. Historically, intense genetic selection for production traits has resulted in a reduction in fertility [2], and although selection traits have broadened in recent years, sub-optimal fertility remains one of the principal reasons for premature culling of cows [3]. However, the role of the bull in determining successful pregnancy outcomes is often overlooked. Despite extensive *in vitro* quality control checks of semen prior to its use in artificial insemination (AI), individual bull’s pregnancy rates can differ by up to 30% [4]. Various analytical techniques, such as computer assisted sperm analysis (CASA) and flow cytometry, have been used to elucidate variation in semen quality, with consequences for bull fertility. With these methods, although 47% of the variation in bull fertility in the field can now be explained by *in vitro* evaluations [5,6], a large component of bull fertility remains unaccounted for. Given that a single bull used in AI can inseminate thousands of females per season, it is clear that optimal fertility of the sire is of critical importance to herd fertility outcomes [4].

The reasons for differences in fertility between AI bulls are likely to be multifactorial, involving issues with sperm transport in the female reproductive tract, fertilization, pre-implantation embryonic development and conceptus elongation as well as development of the embryo and placenta after maternal recognition of pregnancy [7]. Understanding the molecular mechanisms involved in sperm maturation, and between sperm and the female reproductive tract underpinning sperm-oviduct and uterine interactions, will involve identifying proteins which determine fertility outcomes. These proteins may improve diagnosis of infertility as well as identify targets for enhanced breeding in livestock.

Sperm are coated with many different multifunctional peptides, including β-defensins. β-defensins are peptides defined by a conserved six-cysteine motif, which forms three disulphide bridges in a 1-5, 2-4, 3-6 arrangement [8]. Some β-defensins are short – typically ~60 amino acids in length, while others have an extended C-terminal tail, containing glycosylation sites [9,10]. Initially characterised as antimicrobial peptides, β-defensins also have important roles as inflammatory mediators and in male reproductive traits. For example, male mice with deletions of β-defensin genes exhibit infertility [11], and a variant of β-defensin 126 is associated with infertility and reduced sperm motility in men [12,13]. The functional role of β-defensin 126 has also been explored in macaques, where it is secreted in the epididymis, and binds to sperm with the heavily-glycosylated C-terminal tail which forms part of the negatively-charged sperm glycocalyx [14]. It facilitates penetration of cervical mucus and mediates attachment of the sperm to oviduct epithelia [14–16]. In cattle, β-defensin 126 is adsorbed on the tail and post-acrosomal region of the sperm and promotes sperm motility [17]. In addition, β-defensins may also impact on the regulation of the immune response in the female reproductive tract to improve sperm survival and/or pregnancy outcomes. A study directly comparing the *in vivo* uterine response of sperm from bulls with different fertility phenotypes showed an upregulation of genes associated with pregnancy establishment in response to sperm from the high fertility bulls [18], although the role of β-defensins was not assessed.

The β-defensin gene family are known to show recent duplications and genetic divergence, forming clusters of often-closely related genes. Several mammalian lineages show evidence of multiple gene copies – known as copy number variation (CNV), in β-defensin genes between individuals [19]. This observation includes cattle [20,21], where *DEFB103*, in particular, shows extensive multiallelic CNV and shows a diploid copy number (CN) range from 1 up to 29 gene copies across seven different cattle breeds [20]. Studies of β-defensin genes in cattle [22] and rats [23] have shown regional expression patterns of β-defensins in the epididymis. In rats, *Defb14*, an ortholog of *DEFB103*, is expressed in both the testis and cauda epididymis [23]. We have found that *DEFB103*, which encodes BBD103, is expressed in the caput of the epididymis in cattle; is upregulated in testes during sexual maturation from calves to bulls and that mRNA expression levels negatively correlate with gene CN [20]. Taken together, we hypothesize that *DEFB103* CNV is a strong genetic candidate for affecting bull fertility, with the encoded BBD103 protein potentially influencing fertility outcomes and related phenotypes.

**Materials and Methods**

**Ethical approval**

### The procedures were established in compliance with the Cruelty to Animals Act (Ireland 1876, as modified by European Communities regulations 2002 and 2005) and the European Community Directive 86/609/EC.

### Reagents

Unless specified, all chemical substances were obtained from Merck, Arklow, Co Wicklow, Ireland.

**Bull selection**

Data on the field fertility of a population of Holstein-Friesian bulls (n = 840) used in Ireland were obtained from the Irish Cattle Breeding Federation (ICBF) database. Each bull had a minimum of 500 inseminations based on an adjusted sire fertility model [24]. Adjusted bull fertility was defined as pregnancy to a given service identified retrospectively either from a calving event or where a repeat service (or a pregnancy scan) deemed the animal not to be pregnant to the said service. Cows and heifers that were culled or died on farm were omitted. These raw data were then adjusted for factors including semen type (frozen, fresh), cow parity, days in milk, month of service, day of the week when serviced, service number, cow genotype, herd, AI technician, and bull breed and were weighted for number of service records resulting in an adjusted pregnancy rate centered at 0%. For this study, bulls classified as having HF had an average adjusted fertility score of +6.5 whereas, those classified as LF was -6.6%. Genotyping data of bulls for *DEFB103* CNV was previously obtained [20] using digital droplet PCR (ddPCR). Among 20 Holstein Friesian bulls, 9 LF sires were categorized based on their *DEFB103* CNV genotype (into three groups: low CN (n=3 bulls (the lowest CN among the four bulls)), intermediate CN (n=3 bulls), and high CN (n=3 bulls)). In the *DEFB103* genotype, which we previously defined as multiallelic, a diploid CN of 6 was used as the reference, and distinct threshold values of <4.5 and >7.5 were used to define low CN and high CN of this gene, respectively. Among LF bulls with low, intermediate and high CNV, mean adjusted pregnancy rates were -8.96%, -3.96% and -7.8%, respectively (Table 1).

**Table 1:** **Individual-specific data on copy number variation (CNV) for the DEFB103 gene across all three loci (including DEFB103A, DEFB103A-Like, and DEFB103B) and fertility phenotypes namely, low fertility (LF) and high fertility (HF) in Holstein Friesian bulls (n=10 per group).** Evaluating the combined loci of DEFB103, the classification of CNV for the diploid genome categorizes them as having low, intermediate, or high.

| **Individual bull ID** | **Fertility Status** | **Adjusted Pregnancy Rate (%)** | **Number of Inseminations which the Adjusted Pregnancy Rate is based on** | ***DEFB103* CN** | ***DEFB103* CNV**  **Status** |
| --- | --- | --- | --- | --- | --- |
| H1 | HF | 6.8 | 100288 | 2.22 | Low |
| H2 | HF | 6.5 | 3912 | 3.04 | Low |
| H3 | HF | 5.8 | 11459 | 2.76 | Low |
| H4 | HF | 6.8 | 12424 | 5.82 | Intermediate |
| H5 | HF | 7.1 | 34973 | 2.34 | Low |
| H6 | HF | 6.2 | 17441 | 3.09 | Low |
| H7 | HF | 6.7 | 1041 | 2.94 | Low |
| H8 | HF | 6.2 | 5119 | 4.38 | Low |
| H9 | HF | 6 | 8637 | 2.13 | Low |
| H10 | HF | 6.7 | 37849 | 5.82 | Intermediate |
| L1 | LF | -8.5 | 1034 | 2.19 | Low |
| L2 | LF | -4.3 | 23811 | 6.42 | Intermediate |
| L3 | LF | -3 | 568 | 6.93 | Intermediate |
| L4 | LF | -9.1 | 740 | 3.51 | Low |
| L5 | LF | -4.6 | 597 | 5.43 | Intermediate |
| L6 | LF | -3.5 | 1195 | 4.35 | Low |
| L7 | LF | -9.3 | 519 | 3.69 | Low |
| L8 | LF | -12.3 | 1477 | 13.05 | High |
| L9 | LF | -7.3 | 1772 | 19.35 | High |
| L10 | LF | -3.9 | 980 | 7.68 | High |

Sperm physiology data were provided by Stiavnicka et al., [25] and were used to examine the effect of *DEFB103* CNV on various sperm motility and kinematic parameters (assessed using CASA), as well as functional traits including viability, membrane fluidity, and acrosomal status (assessed using flow cytometry) *in vitro* under both capacitating and non-capacitating conditions. These data were generated based on three straws per ejaculate (3 ejaculates per bull) in *in vitro* experiments. Modified Tyrode’s medium (mTALP) was used to create the appropriate conditions for capacitation [26], which contains key ingredients such as NaHCO3, CaCl2, BSA and Heparin, and non-capacitation medium (NCM), is similar but lacks these ingredients, containing increased NaCl and polyvinyl alcohol. Frozen-thawed sperm were washed to remove the egg yolk-based extender through centrifugation at 300×g for 5 min and resuspended in 200 μL NCM, followed by a second centrifugation. The sperm pellet was then resuspended in either NCM or mTALP, with the concentration adjusted to 15 × 10⁶ sperm per mL using a haemocytometer. Sperm in mTALP were incubated at 38.5°C with 5% CO2 for 4 h (capacitating conditions), while sperm in NCM were maintained at room temperature (non-capacitating conditions). To induce the acrosome reaction, Calcium Ionophore A23187 (CaI), along with a dimethyl sulfoxide (DMSO) vehicle control were used. A corresponding vehicle control with DMSO was included, and both samples were incubated at 37°C for 1 h.

**Ability of sperm from bulls with different *DEFB103* CNV genotypes to swim through artificial mucus**

Sperm from two straws from each low fertility bull (n=9) were diluted using a hemocytometer to attain a final sperm concentration of 10 x 10^6^ per mL in TALP medium, which included Hoechst 33342 fluorescent dye (10 mg/mL in 2.3% sodium citrate). This mixture was then incubated at 37°C for 5 min to remove semen extender. The ability of sperm to penetrate cervicovaginal mucus was assessed using the artificial mucus method previously described by Al Naib et al.[27]. Briefly, artificial mucus was formulated by diluting a sodium hyaluronate solution (MAP-5, Lab-stock MicroServices, Ireland) with phosphate-buffered saline, resulting in a final concentration of 6 mg of sodium hyaluronate per mL. Flattened capillary tubes (measuring 0.3 mm x 3.0 mm x 100 mm; Composite Metal Services Ltd, UK) were marked at 10 mm intervals from 10 to 90 mm. These tubes were subsequently filled with artificial mucus and sealed at one end. Two vertically positioned capillary tubes were placed in a 1.5 mL Eppendorf tube containing Hoechst-stained sperm. Thus, for each replicate, each bull sperm was represented by two capillary tubes. The tubes were incubated at 37°C for 1 h, then placed on a hotplate at 45°C for 1 min to immobilize the sperm. Sperm were counted across the width of the tube within one field of view wide, at 10 mm intervals using a fluorescent microscope (10x; Olympus BX 60).

**Ability of sperm from bulls with different *DEFB103* CNV genotypes to bind to oviductal explants *in vitro***

The *in vitro* oviductal epithelium binding capacity of sperm from three different ejaculates from low-fertility bulls, characterized by the *DEFB103* CNV genotype (low CN (n=3), intermediate CN (n=3) and high CN (n=3)) was performed as per the method outlined by Lyons et al. [16]. Each bull's thawed semen was diluted with a prepared stock culture containing medium 199 (M199) supplemented with fetal bovine serum (10%) and gentamicin sulfate (0.25 mg/mL). The sperm concentration was adjusted to 5 x 10^6^ sperm per mL. Reproductive tracts were collected from non-pregnant heifers (n = 9) at a commercial abattoir. Various stages of the oestrous cycle were represented, as previous literature has indicated that the stage of the oestrous cycle does not impact sperm binding *in* vitro [28]*.* Tissue explants were taken from the isthmic portion of the oviduct, and the explants from both oviducts were combined for each individual tract. Explants were prepared by cleaning oviducts, isolating the isthmic segment, and releasing epithelial cells through gentle compression and fragmentation. These were cultured in M199 medium supplemented with fetal calf serum and gentamicin sulfate, incubated to form everted vesicles, and used within 5 h of slaughter. For the binding assay, explants were combined with sperm (5 X 10^6^ sperm per mL) pre-stained with Hoechst 33342 dye for enhanced binding visualisation [16]. After incubation and removal of loosely bound sperm by gently pipetting through two 75 μL droplets of M199 media, samples were examined under a fluorescent microscope. A total of ten explants from each treatment were randomly evaluated, and the density of sperm binding determined by calculating the number of bound spermatozoa per 0.1 mm^2^ of explant surface.

***In vivo* sperm migration into the oviduct and uterine transcriptomic response in bulls with different *DEFB103* CNV genotypes**

*Heifer selection*

Eighteen 24- to 30-month-old cross-bred heifers (Charolais, Limousin, Aberdeen Angus and Simmental) had their oestrus cycles synchronized and were inseminated with 15 x 10^6^ sperm following a fixed-time AI protocol. The heifers were blocked by breed and age into 3 distinct treatment groups and inseminated with semen from 3 different genotype bull group each characterized by low CN (2 heifers per bull), intermediate CN (2 heifers per bull), and high CN (2 heifers per bull). To maintain consistency, 2 semen straws, each derived from separate ejaculates, were used for insemination from each bull. However, a complication arose on the day of AI, necessitating an alteration in the procedure. Specifically, while 6 crossbred heifers will be used for each bull genotype group; 7 crossbred heifers with Low CN, 6 crossbred heifers with Intermediate CN and 5 crossbred heifers with High CN and were inseminated with frozen-thawed sperm from the respective bulls. The synchronization protocol entailed a seven-day intravaginal progesterone device (CIDR®), accompanied by intramuscular administration of gonadotrophin-releasing hormone (Ovarelin; 2 mL) at CIDR® insertion. Prostaglandin F2 alpha (Enzaprost; 5 ml) was administered intramuscularly to induce luteolysis 24 h before CIDR removal. At 54 (+/- 1) h post-CIDR® removal, heifers received a single fixed-time insemination of frozen–thawed semen. Ovarelin (2 mL) was administered intramuscularly at the time of AI. Heifers were re-evaluated after slaughter for ovulation based on the absence of the corpus luteum as well as the presence of either a large pre-ovulatory follicle or a fresh ovulation [29], and it was confirmed that all heifers were in the follicular phase of their cycle.

*Tissue collection and RNA extraction*

Twelve hours post insemination, heifers were slaughtered at a commercial abattoir. Reproductive tracts were promptly collected post-mortem, and the uterine horn on the side of the ovulated ovary was longitudinally opened with sterile scissors. Tissue samples were acquired from the inter-caruncular region of the uterine horn near the uterine body using a sterile 8-mm biopsy punch. The endometrium was then dissected away from the myometrium. Endometrium samples were flash frozen in liquid nitrogen, transported to the laboratory, and stored at −80°C.

Total RNA isolation, RNA library preparation and sequencing was conducted by Biomarker Technologies (BMKgene, Munster, Germany). Paired-end (150bp) sequencing was performed on an Illumina Novaseq 6000 sequencer to a raw read depth of approximately 50 million total reads (303.20 Gb) per sample. Percentage of Q30 bases in each sample was above 98.18 %.

*Quality control, mapping, and differential read count quantification*

Raw sequence reads were obtained in FASTQ format and their quality was evaluated using FastQC (v.0.12.1). The sequences from all samples were then quality-trimmed and cleared of adaptor sequences using the BBDuk Java package. For alignment, trimmed reads were semi-mapped to the bovine reference genome (ARS-UCD1.2) using Salmon [30], and uniquely mapped read counts per Ensembl (version 104) annotated gene/transcript were estimated using the Salmon–quant mode option. To ensure robustness, only genes with a minimum of five reads across all samples were considered in subsequent analyses. MultiQC analysis was then conducted to derive essential statistics on the percentage of reads counted in the transcriptome. The Tximport function (v.1.3.9) [31] in R 4.3.1 summarized the results at the per-transcript gene level.

Differential gene expression analysis, along with data transformations and visualization, was performed using DeSeq2 (v3.18) [32] in R 4.3.1. Samples were clustered based on variance-stabilizing transformed data and visualized through principal component analysis (PCA). Differentially expressed gene (DEG) lists were created using a negative binomial generalized linear model, and comparisons were conducted among uterine biopsy groups. P-values were adjusted for multiple comparisons using the Benjamini and Hochberg (B–H) method. Genes with an adjusted P-value (False Discovery Rate (FDR)) <0.1 were considered differentially expressed and used for further data exploration and pathway analysis.

Gene Ontology (GO) and Kyoto Encyclopedia of Genes and Genomes (KEGG) pathway analysis were used for all DEG regardless of specific *DEFB103* CN groups. GO term was analyzed using the Gene Ontology knowledge base [33,34]. In the pathway analysis, the analysis of DEGs was performed using ClusterProfiler (v3.18.0) based on the KEGG database [35]. In this analysis, GO terms and pathway analysis were defined as P-adjusted (FDR) ≤ 0.05.

**Recovery of sperm from bovine oviduct**

Oviducts from the 18 heifers (total n = 36 paired oviducts) were collected and the ipsilateral and contralateral oviducts flushed for sperm separately. One end of each oviduct was tied with a cotton thread, spTALP solution with heparin (10 μg/mL) was prepared and injected into the oviducts with a volume of average 2 ml, and the oviducts were closed. After 60 min of incubation, the oviducts were milked, the volume assessed and sperm concentration was assessed in supplicate using a haemocytometer.

**Statistical analysis**

Pearson correlation coefficients were calculated for the relationship between *DEFB103* CNV with CASA and Flow Cytometry data, and a linear regression analysis was performed between the results via GraphPad Prism (v 9.5.1). All other data were checked for normality of distribution and analyzed by univariate analysis of variance (ANOVA) with Bonferroni post-hoc tests in GraphPad Prism (v 9.5.1). Statistical significance level was set at p ≤0.05. All results are presented as mean ± standard error of the mean.

**Results**

**Impact of *DEFB103* CNV on sperm motility, mucus penetration, and oviductal epithelial binding**

The objective of the first part of the study was to assess the impact of *DEFB103* CNV on sperm functional parameters, mucus penetration, and binding to the oviduct epithelium. Across all bulls, *DEFB103* CN was strongly negatively associated with total motility (p<0.05) and progressive motility (*p*<0.05) [Table 2]. Low *DEFB103* CN was associated with lower straight-line velocity (VSL; *p*<0.05), average path velocity (VAP; *p*<0.05), and higher sperm-oviduct binding capacity (*p*<0.05; Table 3; Figure 1). Moreover, the percentage of viable sperm with high membrane fluidity in *in vitro* non-capacitating conditions demonstrated that high *DEFB103* CN was associated with higher membrane fluidity compared to the intermediate CN group (*p*<0.05). There was no effect of *DEFB103* CN on the ability of sperm to penetrate artificial mucus.

**Figure 1:** **The ability of frozen-thawed sperm from low fertility Holstein-Friesian bulls with varying *DEFB103* copy number variation (CNV) to bind to the oviductal epithelial *in vitro.*** Mean and standard errors of three replicates are shown for low fertility bulls (n=9). The central slope indicates the regression curve obtained through linear regression analysis. r=-0.5, p=0.006.

**Table 2:** **Correlation between *DEFB103* copy number variation (CNV) and sperm functional parameters in Holstein-Friesian bulls with high (HF) and low fertility (LF) phenotypes (n=10 per group).** P values <0.05 are shown in bold.

| Parameter | Pearson r | *P* value |
| --- | --- | --- |
| Computer Assisted Sperm Analysis (CASA) |  |  |
| Total motility (%) | -0.64 | **0.003** |
| Progressive motility (%) | -0.54 | **0.015** |
| Curvilinear velocity (VCL; µm/s) | 0.07 | 0.76 |
| Straight-line velocity (VSL; µm/s) | 0.33 | 0.16 |
| Average path velocity (VAP; µm/s) | 0.29 | 0.21 |
| Hyperactivation (%) | -0.34 | 0.14 |
| Flow cytometry |  |  |
| Viable (%) in capacitating conditions | -0.29 | 0.21 |
| Acrosome reacted (%) in capacitating conditions | -0.21 | 0.38 |
| Acrosome intact (%) in capacitating conditions | 0.20 | 0.38 |
| High membrane fluidity (%) in capacitating conditions | -0.13 | 0.57 |
| Viable (%) in non-capacitating conditions | -0.18 | 0.44 |
| Viable, acrosome react (%) in non-capacitating conditions | 0.01 | 0.94 |
| Viable, acrosome intact (%) in non-capacitating conditions | -0.01 | 0.94 |
| Viable, high membrane fluidity (%) in noncapacitating conditions | -0.06 | 0.78 |

**Table 3: Effect of *DEFB103* copy number variation (CNV) on sperm functional parameters assessed using computer assisted sperm analysis (CASA), flow cytometry, mucus penetration and sperm binding to oviductal epithelial cells *in vitro* in low fertility (LF) Holstein-Friesian bulls.** Values are mean ± s.e.m. n=3 bulls per group. P values <0.05 are shown in bold.

| **Parameter** | **CNV status** | | | | **Comparisons of each CN group (*P* value)** | | |
| --- | --- | --- | --- | --- | --- | --- | --- |
|  | **Low** | **Intermediate** | | **High** | **High vs Low** | **High vs Intermediate** | **Low vs Intermediate** |
| **CASA** |  |  |  | |  |  |  |
| Total motility (%) | 57.2±4.39 | 50.6±5.75 | 47.2±4.56 | | 0.82 | >0.99 | >0.99 |
| Progressive motility (%) | 42.4±4.32 | 40.4±5.66 | 36.9±4.49 | | >0.99 | >0.99 | >0.99 |
| Curvilinear velocity (VCL; mm/s) | 89.7±6.70 | 107.9±5.20 | 103.7±9.16 | | 0.42 | >0.99 | 0.21 |
| Straight-line velocity (VSL; mm/s) | 58.9±4.98 | 80.7±5.02 | 79.0±9.49 | | 0.11 | >0.99 | **0.04** |
| Average path velocity (VAP; mm/s) | 69.9±4.42 | 90.7±4.62 | 84.1±8.67 | | 0.14 | 0.58 | **0.03** |
| Hyperactivation (%) | 6.8±3.31 | 3.7±1.11 | 4.5±2.39 | | >0.99 | >0.99 | >0.99 |
| **Flow cytometer** |  |  |  | |  |  |  |
| Viable (%) in capacitating conditions | 14.2±3.90 | 10.3±3.86 | 14.4±3.02 | | 0.6 | 0.99 | 0.57 |
| Acrosome reacted (%) in capacitating conditions | 10.1±3.12 | 15.1±4.10 | 5.2±1.19 | | 0.79 | 0.09 | 0.77 |
| Acrosome intact (%) in capacitating conditions | 89.8±3.12 | 84.8±4.10 | 94.8±1.19 | | 0.79 | 0.09 | 0.77 |
| High membrane fluidity (%) in capacitating conditions | 13.1±3.91 | 5.6±1.31 | 8.6±1.32 | | 0.29 | 0.56 | 0.051 |
| Viable (%) in non-capacitating conditions | 21.0±4.11 | 16.0±1.62 | 21.1±2.51 | | >0.99 | 0.44 | 0.47 |
| Acrosome reacted (%) in non-capacitating conditions | 1.7±0.82 | 2.8±1.32 | 1.0±0.11 | | >0.99 | 0.49 | >0.99 |
| Acrosome intact (%) in non-capacitating condition | 98.2±0.85 | 97.1±1.33 | 98.9±0.11 | | >0.99 | 0.49 | >0.99 |
| High membrane fluidity (%) in non-capacitating conditions | 3.7±1.42 | 2.4±12 | 5.6±0.90 | | 0.32 | **0.003** | 0.17 |
| **Mucus penetration (number of sperm)** | 113.5±13 | 122.0±59 | 122.5±22.93 | | 0.94 | 0.73 | 0.55 |
| **Sperm binding to oviductal epithelium (per 0.1 mm^2^)** | 1.6±0.18 | 1.0±0.08 | 1.1±0.04 | | 0.051 | 0.97 | **0.03** |

**Effect of *DEFB103* copy number variation on sperm migration to the oviducts and the uterine transcriptomic response**

Given the effect of low CNV on sperm phenotype, in particular on sperm oviductal binding, the effect of the sperm from bulls with different *DEFB103* CN on sperm migration to the oviducts and uterine response *in vivo* was investigated. The same number of sperm were inseminated into each heifer (15 x 10^6^) and an average of 0.16 x 10^6^ sperm were recovered from the contralateral oviduct and 0.34 x 10^6^ sperm were recovered from the ipsilateral oviduct. There was no difference (p>0.05) in the number of sperm recovered from the ipsilateral and contralateral oviducts and a large variation between individual animals was observed. Similarly, no relationship between sperm concentration recovered by flushing and the *DEFB103* CN of the bull was detected (Figure 2).

**Figure 2.** **The ability of frozen-thawed sperm from low fertility** **Holstein-Friesian bulls (n=3 per group) with varying *DEFB103* copy number to populate the oviduct *in vivo*.** (A) Sperm recovered from the contralateral oviduct, and (B) Sperm recovered from the ipsilateral oviduct after flushing. The X-axis represents the genotype, while the Y-axis indicates the sperm concentration (x 10^6^).

Principal component analysis of global transcript levels from the 18 different heifers uterine tissue showed no clear clustering based on the *DEFB103* CN of the bull whose semen was used for insemination (Supplementary Table S1) indicating an absence of global differences due to sperm treatment. This is likely accounted for by the complexity of diverse sperm-uterine interactions occurring simultaneously as well as the heterogeneity due to the use of different heifers. To identify more subtle differences between treatments, three pairwise comparisons were performed: 1. between heifers inseminated with semen from high *DEFB103* CN bulls and those inseminated by low CN bulls; 2. between heifers inseminated with semen from high *DEFB103* CN bulls and those inseminated by intermediate CN bulls; and 3. between heifers inseminated with semen from intermediate *DEFB103* CN bulls and those inseminated by low CN bulls.

A total of 58 genes were differentially expressed in the uterus post-insemination using sperm from *DEFB103* bulls using a threshold of FDR<0.1 (Full list shown in Supplementary Table S1). Some of these genes had a low log_2_ fold change (FC) and those with a log_2_FC≤-2 or are shown in Table 4. In comparison 1, the high CN group showed increased expression of 5 genes (*KYAT3, CADM3, SEMA3C, SEMA3A*, and *SLC35F1*) compared to the low CN group, while the expression of 6 genes (*SKAP2, RPS3A, TGIF1*, *EFR3B,* and *CYP2C87*), including Calponin 3 (*CNN3*), was decreased in same comparison.

**Table 4 Differential gene expression analysis results comparing intermediate copy number (CN) bulls versus low CN bulls, low CN bulls versus high CN bulls and intermediate CN bulls versus high CN bull.** Genes with statistically significant differences in expression (FDR<0.1) and log_2_ fold changes <-2 or >+2 were filtered for inclusion in the table.

| **Gene Symbol** | **Description** | **Gene ID** | **Log_2_** **FC** | **FDR** |
| --- | --- | --- | --- | --- |
| **Sperm from High CN versus Low CN bulls** | | | | |
| *CNN3* | Calponin 3 | ENSBTAG00000024407 | -9 | 6.70x10^^-02^ |
| *RPS3A* | Ribosomal Protein S3A | ENSBTAG00000006383 | -8.46 | 4.10x10^^-02^ |
| *CYP2C87* | Cytochrome P450, family 2, subfamily C, polypeptide 87 | ENSBTAG00000037795 | -2.6 | 9.00x10^^-02^ |
| **Sperm from High CN** **bulls versus Intermediate CN bulls** | | | | |
| *RPS3A* | Ribosomal Protein S3A | ENSBTAG00000006383 | -9.18 | 3.80x10^-03^ |
| *BRINP3* | BMP/retinoic acid inducible neural specific 3 | ENSBTAG00000008420 | 5.53 | 8.10x10^-02^ |
| *CDH9* | cadherin 9 | ENSBTAG00000018234 | 7.46 | 1.20x10^-03^ |
| *RPS4* | Small ribosomal subunit protein | ENSBTAG00000031205 | -7.86 | 3.30x10^-03^ |
| *ITLN2* | intelectin 2 | ENSBTAG00000048662 | -6.14 | 4.90x10^-02^ |
| *GABRA4* | Gamma-aminobutyric acid type A receptor subunit alpha4 | ENSBTAG00000016645 | 4.78 | 6.30x10^-02^ |
| *PRRT3* | Proline rich transmembrane protein 3 | ENSBTAG00000010836 | 3.08 | 1.30x10^-02^ |
| *SAA* | Serum amyloid A protein | ENSBTAG00000022395 | -2.04 | 4.90x10^-02^ |
| *AKR1C1* | Aldo-keto reductase family 1, member C1-like | ENSBTAG00000037509 | -2.69 | 3.00x10^-02^ |
| *GRO1* | Chemokine (C-X-C motif) ligand 1 (melanoma growth stimulating activity, alpha) | ENSBTAG00000037558 | -3.55 | 6.90x10^-02^ |
| *CXCL3* | Chemokine (C-X-C motif) ligand 3 | ENSBTAG00000037778 | -2.39 | 6.90x10^-02^ |
| *NRXN1* | Neurexin 1 | ENSBTAG00000046199 | 2.11 | 5.70x10^-02^ |
| **Sperm from Intermediate CN bulls versus Low CN bulls** | | | | |
| *RPS4* | Small ribosomal subunit protein | ENSBTAG00000031205 | 8.58 | 1.70x10^-04^ |
| *CNN3* | Calponin 3 | ENSBTAG00000024407 | -8.47 | 6.50x10^-02^ |
| *GRO1* | Chemokine (C-X-C motif) ligand 1 (melanoma growth stimulating activity, alpha) | ENSBTAG00000037558 | 3.67 | 6.50x10^-02^ |
| *CXCL3* | Chemokine (C-X-C motif) ligand 3 | ENSBTAG00000037778 | 2.54 | 6.50x10^-02^ |

The greatest difference in transcriptomic response was detected when comparing uterine responses to sperm from high and intermediate CN bulls (41 genes Log_2_FC>2, Full list shown in Supplementary Table S1). There was a decrease in the expression of 12 genes, especially *RPS3A, RPS4, AKR1C1, GRO1, CXCL3*, and *ITLN2,* in the high CN group compared to the intermediate CN group. In contrast, among the up-regulated genes, *BRINP3, PRRT3, GABRA4*, and *CDH9* showed the largest increases in their expression levels in response to sperm in the uterine endometrium. In comparison 3, the intermediate CN group was compared with the low CN group, a total of 6 genes were differentially expressed. Among these, *CNN3* showed an almost 8.5-fold lower expression, while *GRO1, CXCL3,* and *RPS4* showed significantly higher expression.

Gene ontology analysis of DEG, identified in the three pairwise comparisons, indicated cell migration as a key process influenced by *DEFB103* CN of the bull (Table 5A). Kyoto Encyclopedia of Genes and Genomes (KEGG) analysis identified several potential pathways affected – of particular interest was cell adhesion processes and the *NFKBIA/GRO1/CXCL3* signalling axis (Table 5B). Low *DEFB103* CN in bulls was associated with increased expression of genes promoting uterine preparation for embryonic development and chemotaxis. Conversely, high *DEFB103* CN was associated with the high expression of genes affecting cell adhesion and proliferation, and with low expression of genes that are crucial for protein synthesis and immune response, potentially affecting these processes in the uterine environment (all details shown in Supplementary Table S1).

**Table 5A: Gene Ontology (GO) analysis for heifers inseminated with frozen-thawed semen from low fertility bulls, categorized by different *DEFB103* genotypes.** This analysis includes all differentially expressed genes (DEGs) with a corrected p-value of less than 0.01.

| **GO: Biological process** | | | | |
| --- | --- | --- | --- | --- |
| **Category** | **GO ID** | **Gene** | **Definition** | ***P-*value** |
| Neural crest cell migration | GO:0001755 | *SOX8/TWIST1/SEMA3C/SEMA3A* | The characteristic movement of cells from the dorsal ridge of the neural tube to a variety of locations in a vertebrate embryo | 0.8x10^-02^ |
| Mesenchymal cell migration | GO:0090497 | *SOX8/TWIST1/SEMA3C/SEMA3A* | The orderly movement of a mesenchymal cell from one site to another, often during the development of a multicellular organism | 0.6x10^-02^ |

### Table 5B: Analysis of the top 5 significant KEGG pathways for heifers inseminated with frozen-thawed semen from low fertility bulls, categorized by different *DEFB103* genotypes. This analysis includes all differentially expressed genes (DEGs) with a corrected p-value of less than 0.01.

| **KEGG ID** | **Category** | **Description** | **Gene in pathway** | **P-value** |
| --- | --- | --- | --- | --- |
| bta04514 | Signalling molecules and interaction | Cell adhesion molecules | *CADM3/LRRC4B/NRXN1/BOLA* | 7.20x10^-04^ |
| bta04360 | Development and regeneration | Axon guidance | *ROBO1/ROBO2/SEMA3A/SEMA3C* | 1.02x10^-03^ |
| bta04657 | Immune system | IL-17 signalling pathway | *NFKBIA/GRO1/CXCL3* | 1.60x10^-03^ |
| bta04064 | Signal transduction | NF-kappa B signalling pathway | *NFKBIA/GRO1/CXCL3* | 2.60x10^-03^ |
| bta04668 | Signal transduction | TNF signalling pathway | *NFKBIA/GRO1/CXCL3* | 3.90x10^-03^ |

**Discussion**

In this study, we investigated the effect of bull *DEFB103* CNV on sperm motility, kinematic parameters and functional parameters in Holstein-Friesian bulls. Across all bulls, *DEFB103* CN was strongly negatively associated with total motility and progressive motility (-0.6 and -0.5, respectively), potentially explaining a proportion of the variation in fertility between these groups. This builds on previous work showing that *DEFB103* is expressed in the caput epididymis, extensive multiallelic CNV negatively correlates with its expression level in the testis and it is upregulated at sexual maturity [20]. Consequently, reduced expression of *DEFB103* protein may impair sperm motility and function, which may explain the lower progressive and overall motility observed in bulls with higher *DEFB103* CN.

Due to inadequate CNV diversity in HF bulls, the remainder of the study specifically examined sperm progression to the oviducts *in vivo* and the uterine transcriptomic response to sperm in LF bulls only. Within the LF group, a statistically significant effect on two critical sperm kinematic parameters - VSL and VAP, with sperm from low CV bulls displaying reduced velocity. The observed disparities in sperm kinematic parameters suggest that high or low fertility cannot be attributed solely to *DEFB103* CN and it is possible that an additive effect across the β-defensin gene locus could have a stronger effect on the overall fertility phenotype.

Upon entering the female reproductive tract, sperm undergo crucial membrane transformations that are essential for fertilization [36]. Previous research by Stiavnicka *et al.*,[25] showed that LF bull sperm had reduced membrane fluidity under *in-vitro* capacitating conditions compared to HF bulls. Results from the current study suggests that high *DEFB103* increases membrane fluidity, especially under capacitating conditions, may facilitate stronger binding to the oviductal epithelium. A number of studies have shown differences in the ability of sperm to bind to the oviductal epithelium between HF and LF bulls [7]. Considering β-defensins are known to play a critical role in this process [16,37], results from this study illustrated that high *DEFB103* CN reduced the oviductal binding of sperm suggesting that lower *DEFB103* CN might improve fertility outcomes by enhancing sperm binding capabilities.

After demonstrating a clear effect of *DEFB103* indirectly on sperm function, we sought to determine whether the uterine response varied depending on the sperm from bulls with different *DEFB103* CN. Overall, transcriptomic differences were modest, but *DEFB103* CN had significant effects on expression of genes with roles in sperm migration, immune signalling, and chemotaxis pathways. *RPS3A*, which is an integral part of ribosomal proteins and critical for protein synthesis [38], is involved in actin cytoskeleton regulation, cell adhesion, and tissue remodelling [39], and showed 8.46- and 9.18-log_2_FC decreases in expression levels in the High CN group compared to the Low and Intermediate CN groups, respectively. This reduction may hinder cell adhesion and reorganization, thus posing difficulties for embryo implantation and subsequent development. An almost 9 log_2_FC increase in the expression level of Calponin 3 (*CNN3)* was detected in the Low CN group compared to others. *CNN3* regulates epithelial contractile activity by localizing to actin filaments, and interestingly, studies have shown that this is essential for embryonic development and viability in mice [39]. More importantly, *CNN3*, which showed increased expression in the low *DEFB103* CN group, has been reported to be involved in the cytoskeletal rearrangement required for trophoblastic fusion in the human BeWo cell line, a critical process for embryo implantation and interaction with the decidualized maternal uterus [40], suggesting potential novel regulatory mechanisms of defensins in semen priming the uterus for embryo implantation. *RPS4*, ribosomal protein, along with two chemokines, *GRO1* and *CXCL3*, were significantly upregulated in response to sperm from intermediate CN bulls compared to sperm from the low CN group. *RPS4* plays a role in spermatogenesis [41], suggesting that significant changes in intercellular communication, cell cycle regulation, and protein synthesis occur. *GRO1* has previously been shown to have high expression levels in decidual endometrial stromal cells in response to products secreted by trophoblasts in humans [42]. *CXCL3*, has been shown to play a role in the migration, invasion, proliferation, and tube formation of trophoblast cells [43]. Taken together, these findings suggest that *DEFB103* may contribute to chemotaxis during sperm motility and settlement within the uterus. In contrast, Intelectin 2 (*ITLN2*) expression was 6.14 log_2_FC lower in the high CN group compared to the intermediate CN group. *ITLN2*, plays a role in the innate immune response [44], and may influence immune responses or tissue repair processes in the uterine environment due to its reduced expression in sperm from high CN bulls. There was an increase in Cadherin 9 (*CDH9*) and Gamma-aminobutyric acid receptor subunit alpha-4 (*GABRA4*) expression in sperm from high CN bulls versus intermediate CN bull group. Since *CDH9* plays a role in cell-cell adhesion [45], its increased expression in sperm may increase the interaction between sperm and the uterine epithelium. While this could potentially improve sperm retention and survival in the female reproductive tract; increased expression level of *GABRA4* may negatively affect the preparation of the uterine endometrium for implantation. It has previously been reported that *GABA* and synthetic *GABA* receptor ligands in mice can negatively affect preimplantation embryos through their receptors [46].

Taken together, the findings of this study suggest that *DEFB103* CNV has a significant impact on sperm motility. Furthermore, *DEFB103* CNV exerts a significant impact on LF bulls by affecting various sperm functional parameters and eliciting distinct transcriptomic responses in the uterus post-insemination. Lower *DEFB103* CN, associated with higher *DEFB103* expression levels, likely enhances sperm motility and oviductal epithelium binding capacity by increasing protein availability and affecting charge-mediated progression to the site of fertilisation. Additionally, DEFB103 CN may alter the uterine immune response to sperm from bulls with low-CN, and therefore affecting early pregnancy establishment. These findings illustrate the impact of *DEFB103* CNV on multiple aspects of bovine reproduction and suggests that further dissection of the roles of β-defensins in cattle fertility is now warranted.

**Acknowledgements**

The authors acknowledge Dr Ciara O’ Meara from the National Cattle Breeding Centre for provision of semen straws. We are also grateful to Dr Miriama Stiavnicka for providing CASA and Flow cytometer data for the HF and LF phenotype bulls. We thanks to Kaitlyn Weldon and Nathan Johnston from University of Limerick for help to collect the female reproductive tract samples from the commercial abattoir. This research used the ALICE High Performance Computing facility at the University of Leicester.

**References**

[1]	Hales KE. Relationships between digestible energy and metabolizable energy in current feedlot diets. Transl Anim Sci 2019; 3:945–952.

[2]	Pryce JE, Haile-Mariam M, Goddard ME, Hayes BJ. Identification of genomic regions associated with inbreeding depression in Holstein and Jersey dairy cattle. Genet Sel Evol 2014; 46:71–77.

[3]	Dallago GM, Wade KM, Cue RI, McClure JT, Lacroix R, Pellerin D, Vasseur E. Erratum: Dallago et al. Keeping dairy cows for longer: A critical literature review on dairy cow longevity in high milk-producing countries (Animals, (2021), 11, (808), 10.3390/ani11030808). Animals 2021; 11.

[4]	Fair S, Lonergan P.  Review: understanding the causes of variation in repro- ductive wastage among bulls. Animal 2018; 12:53–62.

[5]	Bernecic NC, Donnellan E, O’Callaghan E, Kupisiewicz K, O’Meara C, Weldon K, Lonergan P, Kenny DA, Fair S. Comprehensive functional analysis reveals that acrosome integrity and viability are key variables distinguishing artificial insemination bulls of varying fertility. J Dairy Sci 2021; 104:11226–11241.

[6]	Sellem E, Broekhuijse MLWJ, Chevrier L, Camugli S, Schmitt E, Schibler L, Koenen EPC. Use of combinations of in vitro quality assessments to predict fertility of bovine semen. Theriogenology 2015; 84:1447-1454.e5.

[7]	Donnellan EM, Lonergan P, Meade KG, Fair S. An ex-vivo assessment of differential sperm transport in the female reproductive tract between high and low fertility bulls. Theriogenology 2022; 181:42–49.

[8]	Cormican P, Meade KG, Cahalane S, Narciandi F, Chapwanya A, Lloyd AT, O’Farrelly C. Evolution, expression and effectiveness in a cluster of novel bovine beta-defensins. Immunogenetics 2008; 60:147–156.

[9]	Narciandi F, Fernandez-Fuertes B, Khairulzaman I, Jahns H, King D, Finlay EK, Mok KH, Fair S, Lonergan P, Farrelly CO, Meade KG. Sperm-Coating Beta-Defensin 126 Is a Dissociation-Resistant Dimer Produced by Epididymal Epithelium in the Bovine Reproductive Tract. Biol Reprod 2016; 95:121.

[10]	Dorin JR. Novel phenotype of mouse spermatozoa following deletion of nine beta-defensin genes. Asian J Androl 2015; 17:716–719.

[11]	Zhou YS, Webb S, Lettice L, Tardif S, Kilanowski F, Tyrrell C, Macpherson H, Semple F, Tennant P, Baker T, Hart A, Devenney P, et al. Partial deletion of chromosome 8 beta-defensin cluster confers sperm dysfunction and infertility in male mice. PLoS Genet 2013; 9:e1003826.

[12]	He J-Y, Peng J-Y, Li Q-F, Lin X-L, Cui Y-R, Ma S-Y, Fan S-Y, Liu Y-R, Song Z-L, Deng J-H, Wei X, Ding X-P. DEFB126 polymorphisms and association with idiopathic asthenozoospermia in China. Asian J Androl 2022; 24:607–614.

[13]	Duan S, Shi C, Chen G, Zheng J, Wu B, Diao H, Ji L, Gu Y, Xin A, Wu Y, Zhou W, Miao M, et al. Another functional frame-shift polymorphism of DEFB126 (rs11467497) associated with male infertility. J Cell Mol Med 2015; 19:1077–1084.

[14]	Tollner TL, Yudin AI, Tarantal AF, Treece CA, Overstreet JW, Cherr GN. Beta-defensin 126 on the surface of macaque sperm mediates attachment of sperm to oviductal epithelia. Biol Reprod 2008; 78:400–412.

[15]	Tollner TL, Venners SA, Hollox EJ, Yudin AI, Liu X, Tang G, Xing H, Kays RJ, Lau T, Overstreet JW, Xu X, Bevins CL, et al. A common mutation in the defensin DEFB126 causes impaired sperm function and subfertility. Sci Transl Med 2011; 3:92ra65.

[16]	Lyons A, Narciandi F, Donnellan E, Romero-Aguirregomezcorta J, Farrelly CO, Lonergan P, Meade KG, Fair S. Recombinant beta-defensin 126 promotes bull sperm binding to bovine oviductal epithelia. Reprod Fertil Dev 2018; 30:1472–1481.

[17]	Fernandez-Fuertes B, Narciandi F, O’Farrelly C, Kelly AK, Fair S, Meade KG, Lonergan P. Cauda epididymis-specific beta-defensin 126 promotes sperm motility but not fertilizing ability in cattle. Biol Reprod 2016; 95.

[18]	Donnellan EM, Cormican P, Colm R, Duggan G, Stiavnicka M, Meade KG, Fair S. The transcriptomic response of bovine uterine tissue is altered in response to sperm from high- and low-fertility bulls. Biol Reprod 2023; 108:912–921.

[19]	Hollox EJ, Abujaber R. Evolution and diversity of defensins in vertebrates. Evolutionary Biology: Self/Nonself Evolution, Species and Complex Traits Evolution, Methods and Concepts. 2017.

[20]	Sidekli O, Oketch J, Fair S, Meade KG, Hollox EJ. β-Defensin gene copy number variation in cattle. R Soc Open Sci 2024; 11.

[21]	Bickhart DM, Hou Y, Schroeder SG, Alkan C, Cardone MF, Matukumalli LK, Song J, Schnabel RD, Ventura M, Taylor JF, Garcia JF, Tassell CP Van, et al. Copy number variation of individual cattle genomes using next-generation sequencing. Genome Res 2012; 22:778–790.

[22]	Narciandi F, Lloyd AT, Chapwanya A, Farrelly CO, Meade KG. Reproductive tissue-specific expression profiling and genetic variation across a 19 gene bovine beta-defensin cluster. Immunogenetics 2011; 63:641–651.

[23]	Patil AA, Cai Y, Sang Y, Blecha F, Zhang G. Cross-species analysis of the mammalian beta-defensin gene family: presence of syntenic gene clusters and preferential expression in the male reproductive tract. Physiol Genomics 2005; 23:5–17.

[24]	Berry DP, Kearney JF. Imputation of genotypes from low- to high-density genotyping platforms and implications for genomic selection. Animal 2011; 5:1162–1169.

[25]	Stiavnicka M, Hosek P, Abril-Parreno L, Kenny DA, Lonergan P, Fair S. Membrane remodulation and hyperactivation are impaired in frozen-thawed sperm of low-fertility bulls. Theriogenology 2023; 195:115–121.

[26]	Parrish JJ, Susko-Parrish JL, First NL. Capacitation of bovine sperm by heparin: Inhibitory effect of glucose and role of intracellular pH. Biol Reprod 1989; 41.

[27]	Al Naib A, Hanrahan JP, Lonergan P, Fair S. In vitro assessment of sperm from bulls of high and low field fertility. Theriogenology 2011; 76.

[28]	Sostaric E, Dieleman SJ, Van De Lest CHA, Colenbrander B, Vos PLAM, Garcia-Gil N, Gadella BM. Sperm binding properties and secretory activity of the bovine oviduct immediately before and after ovulation. Mol Reprod Dev 2008; 75.

[29]	Ireland JJ, Murphee RL, Coulson PB. Accuracy of Predicting Stages of Bovine Estrous Cycle by Gross Appearance of the Corpus Luteum. J Dairy Sci 1980; 63.

[30]	Patro R, Duggal G, Love MI, Irizarry RA, Kingsford C. Salmon provides fast and bias-aware quantification of transcript expression. Nat Methods 2017; 14:417–419.

[31]	Soneson C, Love MI, Robinson MD. Differential analyses for RNA-seq: transcript-level estimates improve gene-level inferences. F1000Res 2015; 4:1521.

[32]	Love MI, Huber W, Anders S. Moderated estimation of fold change and dispersion for RNA-seq data with DESeq2. Genome Biol 2014; 15:550–558.

[33]	Ashburner M, Ball CA, Blake JA, Botstein D, Butler H, Cherry JM, Davis AP, Dolinski K, Dwight SS, Eppig JT, Harris MA, Hill DP, et al. Gene ontology: tool for the unification of biology. The Gene Ontology Consortium. Nat Genet 2000; 25:25–29.

[34]	Consortium GO, Aleksander SA, Balhoff J, Carbon S, Cherry JM, Drabkin HJ, Ebert D, Feuermann M, Gaudet P, Harris NL, Hill DP, Lee R, et al. The Gene Ontology knowledgebase in 2023. Genetics 2023; 224:iyad031. doi: 10.1093/genetics/iyad031.

[35]	Kanehisa M, Goto S. KEGG: kyoto encyclopedia of genes and genomes. Nucleic Acids Res 2000; 28:27–30.

[36]	Travis AJ, Kopf GS. The role of cholesterol efflux in regulating the fertilization potential of mammalian spermatozoa. J Clin Invest 2002; 110:731–736.

[37]	Whiston R, Finlay EK, McCabe MS, Cormican P, Flynn P, Cromie A, Hansen PJ, Lyons A, Fair S, Lonergan P, Farrelly CO, Meade KG. A dual targeted beta-defensin and exome sequencing approach to identify, validate and functionally characterise genes associated with bull fertility. Sci Rep 2017; 7:12287–x.

[38]	Lutsch G, Stahl J, Kargel HJ, Noll F, Bielka H. Immunoelectron microscopic studies on the location of ribosomal proteins on the surface of the 40S ribosomal subunit from rat liver. Eur J Cell Biol 1990; 51:140–150.

[39]	Junghans D, Herzog S. Cnn3 regulates neural tube morphogenesis and neuronal stem cell properties. FEBS Journal 2018; 285.

[40]	Shibukawa Y, Yamazaki N, Kumasawa K, Daimon E, Tajiri M, Okada Y, Ikawa M, Wada Y. Calponin 3 regulates actin cytoskeleton rearrangement in trophoblastic cell fusion. Mol Biol Cell 2010; 21:3973–3984.

[41]	Lopes AM, Miguel RN, Sargent CA, Ellis PJ, Amorim A, Affara NA. The human RPS4 paralogue on Yq11.223 encodes a structurally conserved ribosomal protein and is preferentially expressed during spermatogenesis. BMC Mol Biol 2010; 11.

[42]	Hess AP, Hamilton AE, Talbi S, Dosiou C, Nyegaard M, Nayak N, Genbecev-Krtolica O, Mavrogianis P, Ferrer K, Kruessel J, Fazleabas AT, Fisher SJ, et al. Decidual stromal cell response to paracrine signals from the trophoblast: amplification of immune and angiogenic modulators. Biol Reprod 2007; 76:102–117.

[43]	Wang H, Wang T, Dai L, Cao W, Ye L, Gao L, Zhou B, Zhou R. Effects of CXCL3 on migration, invasion, proliferation and tube formation of trophoblast cells. Placenta 2018; 66:47–56.

[44]	Vazquez CB, Balseiro A, Alonso-Hearn M, Juste RA, Iglesias N, Canive M, Casais R. Bovine Intelectin 2 Expression as a Biomarker of Paratuberculosis Disease Progression. Animals (Basel) 2021; 11:1370. doi: 10.3390/ani11051370.

[45]	Weimin L, Yujing C, Jing L, Zeng H, Ping Z, Enkui D. The expression of CD9 in the peri-implantation mouse uterus is upregulated in an ovarian steroid hormone-dependent manner. Fertil Steril 2007; 87:664–670.

[46]	Kovarikova V, Spirkova A, Sefcikova Z, Pisko J, Kalatova L, Koppel J, Fabian D, Cikos S. Gamma-aminobutyric acid (GABA) can affect physiological processes in preimplantation embryos via GABA(A) and GABA(B) receptors. Reprod Med Biol 2023; 22:e12528.
