# Supplementary material for: Impact of β-defensin 103 (DEFB103) copy number variation on bull sperm parameters and post-insemination uterine gene expression
Source: PLoS One. 2025 Feb 25;20(2):e0319281. doi: 10.1371/journal.pone.0319281 (PMC11856272; doi:10.1371/journal.pone.0319281)
Supplement: Attachment — Submitted filename: Rebuttal-Dec 30th.docx [file pone.0319281.s005.docx]

Dear Dr Ayman A. Swelum,

We would like to thank the reviewers for the comments and suggestions, which have significantly improved the quality of our manuscript. We have addressed all the points raised by the reviewers in the revised version (attached) and below is a point-by-point response. We sincerely hope that the revised version will be considered for publication in *Plos One*.

**Point by point response:**

**Reviewer #1:**

*This is a very good study and was intelligently written. However, there are minor corrections to be made. The authors did infer but did not categorically state the justification for the study, please give a statement to justify the study at the end of the introduction. The pages of the document need to be numbered and authors should ensure consistency in the use of 'et al'. Other comments are highlighted in the document attached.*

***Author response:*** We would like to thank the reviewer for recognising the merit in our study and we have addressed the comments raised by:

1. Justification is now included at end of introduction.
2. Page and line numbers are now added.
3. Checked use of et al.
4. Edited as suggested in previously attached manuscript including substitution of references.
5. We do note however that the opening sentence and reference to seasonal calving is of relevance to the context in which these bulls are used and highlights how sub-optimal bull fertility can have widespread detrimental consequences for pregnancy rates within such a seasonal system, which is unusual in comparison to other dairy systems globally. In grass-based systems, fertility is a key driver of profitability, as there is a need to calve cows compactly at the start of the grass growing season.

**Reviewer #2:**

*General Comments
The manuscript PONE-D-24-51902, titled "Impact of β-defensin 103 (DEFB103) copy number variation on bull sperm parameters and post-insemination uterine gene expression" addresses the potential impact of DEFB103 copy number variation (CNV) on bull fertility traits, including sperm functionality and uterine transcriptomic responses. While the topic is interesting and potentially significant for livestock breeding programs, the manuscript has several critical issues that limit its clarity, scientific rigour, and impact.*

***Author response:*** We would like to thank the reviewer for recognising the relevance of our study topic and the suggestion of useful recommendations. We hope that the following edits address the comments raised.

*Major Concerns
1. Abstract
The abstract focuses heavily on presenting results while providing minimal information about the methodology, making it unbalanced and less informative. Its disjointed structure lacks a smooth flow, resulting in a "jerky" reading experience. The overgeneralization of findings further detracts from its clarity and precision.
Recommendation: A better balance between background, methods, results, and implications is needed to enhance its effectiveness.*

***Author response:*** We have reworked the abstract in line with the reviewers’ comments. We hope now that it is better balanced. We have provided more methodology and in the conclusion we specifically have replaced ‘confirm’ a role with ‘suggest a contributory role for DEFB103 CN’.

*2. Unclear Objectives
The objectives of the study are not clearly stated. While the manuscript appears to explore the effects of DEFB103 CNV on fertility traits, the absence of a concise hypothesis or research question makes it challenging to understand the study's scope and rationale.
Recommendation: Clearly articulate the study objectives and hypothesis in the introduction, ensuring alignment with the methods and results.*

***Author response:*** We have included a new final sentence in the introduction that addresses the objectives of the study.

*3. Introduction: Lack of Focus
The introduction includes extraneous information, such as discussions on cow fertility and seasonal calving systems, which are not directly relevant to the study's focus on bull fertility. Additionally, the rationale for studying DEFB103 CNV is underdeveloped, and the study's novelty is unclear.
Recommendation: Streamline the introduction to focus on:
a. The importance of bull fertility for livestock productivity.
b. Knowledge gaps regarding the role of β-defensins in reproduction.
c. The specific rationale for investigating DEFB103 CNV in bull fertility.*

***Author response:*** The bulls under investigation are widely used in AI, and it is true to state that low fertility bulls used in AI could negatively impact cow fertility, which is exacerbated in seasonal calving systems. We have taken the reviewers comments into account by including additional background details on the importance of bull fertility as well as the relevance of investigating DEFB103 in particular.

*4. Study Design and Methodological Issues
• Sample Size: The small sample size (20 bulls and 18 heifers) limits the statistical power and generalizability of the findings. Additionally, there is no clear explanation of how these bulls were selected from the initially mentioned 840 bulls.
• Methodological Inconsistencies: The decision to focus only on low-fertility bulls for transcriptomic analysis is not well justified, and the study does not adequately control for confounding factors. Moreover, reference no. 28 is incorrectly cited in the methodology section. In actual study cited as reference no.28 is totally opposite than what authors have perceived. It was difficult to understand "why authors decided to use crossbred heifers for Holstein bulls". There too many dependent variables studied on very limited sample size.
Recommendation: Increase the sample size and justify the experimental design more vigorously. Address potential confounders comprehensively.*

***Author response:*** We have included details on how the bulls were selected in the methodology of the revised manuscript. The bulls were selected from the divergent tails of the fertility distribution to maximise the differences in fertility and enhance the prospects of detecting a significant difference due to DEFB103 CNV. Unfortunately, we are not able to increase the sample size as many of these bulls have been replaced by new breeding bulls and semen straws are no longer available. It is also important to note that two straws were used per bull and assays run in duplicate to increase the reproducibility of our findings. This is quite a challenge when working in the laboratory with semen in real time. Reference 28 was incorrectly used and has been replaced. Line 193-196 now reads “Various stages of the oestrous cycle were represented, as previous work by our group (Lyons et al 2018) and others (Lefebvre et al. 1997) have indicated that the stage of the oestrous cycle does not impact sperm binding *in* vitro”. We further emphasise that the *in vivo* work conducted is novel and we acknowledge that there are other study variables that we cannot account for, including heifer breed but these were balanced across CNV treatments. The insemination of crossbred heifers is a standard practice in the farming sector and therefore the findings are relevant and also act as a platform for future more detailed evaluation. In addition, purepbred/pedigree animals are extremely expensive and it is not feasible to perform experiments on these. In addition, purebred animal lack the benefits of heterosis and usually have lower fertility than cross bred females. We have added the following sentence as a caveat in the discussion section ‘Although a constraint of this study is the absence of detailed functional evaluation of CNV within the HF group of bulls, significant phenotypic variation remains within the LF bulls and the findings of this study suggest that *DEFB103* CNV has a significant impact on sperm motility…. Additionally, DEFB103 CN may alter the uterine immune response to sperm from bulls with low-CN, and therefore affecting early pregnancy establishment although further conclusive investigation is required.’. We hope these modifications address the concerns of the reviewer.

*5. Data Interpretation and Overstatement of Findings
The findings, particularly in the transcriptomic analysis, are overstated, given the modest differential expression results (58 genes, FDR < 0.1). Many conclusions lack mechanistic explanations or clear connections to fertility outcomes.
Recommendation: Interpret results more cautiously, emphasizing biologically meaningful findings and acknowledging limitations.*

***Author response:*** An FDR threshold of 0.1 was chosen to ensure a reasonable trade-off between discovering biologically meaningful results and limiting false positives and this cut off has been used in other published transcriptomic studies. We have tried to address the comments of the reviewer by pairing back the claims made and adding the caveats as outlined above. We hope this addresses the suggestions of the reviewer.

*6. Overlap with Previously Published Studies
The manuscript shows substantial overlap with previously published work by the same authors, particularly Sidekli et al. (2024). While including transcriptomic analysis adds some novelty, the manuscript does not adequately differentiate its contributions from prior publications.
Recommendation: Delineate how this study builds upon or diverges from earlier work. Emphasize novel aspects and ensure proper acknowledgement of prior findings.*

***Author response:*** In the previous study we assessed CNV across multiple defensin genes in cattle from a number of populations and did not specifically focus on the role of DEFB103 on bull fertility parameters as assessed here. In addition, the *in vivo* work is novel and although results on differentially expressed genes are modest, it provides a valuable first step to investigate the impact of CNV on the female uterine immune response, which is relevant to pregnancy outcomes. We have emphasised these differences in the revised manuscript.

*7. Discussion and Conclusion
The discussion overstates the significance of findings and lacks integration with existing knowledge on β-defensins, making speculative claims without sufficient data support. It fails to address critical limitations, including the small sample size and lack of estrous cycle control. The conclusion is overly broad, misaligning with the study's modest results and lacking clear practical implications or future research directions.
Recommendation: Reframe the discussion and conclusion to provide balanced interpretations, acknowledge limitations, and suggest realistic next steps.*

***Author response:*** We have redressed the balance as sought by the reviewer. Considering the comments received, we have added multiple caveats (as outlined above). We hope this addresses the suggestions of the reviewer.

*8. Writing Style and Presentation
The writing is dense, and key points are difficult to extract due to overly technical language and a lack of logical flow. Figures and tables lack clarity in presenting results; some are not adequately described in the text. Additionally, the absence of line numbers up to page 22 significantly complicated the review process.
Recommendation: Simplify the language and improve the manuscript's organization for better readability. Revise figures and tables to enhance clarity and alignment with the text. Include line numbers throughout the manuscript for ease of review.
Minor Comments
• References: Replace tangential or outdated references with more recent and directly relevant studies.*

***Author response:*** We appreciate the efforts of the reviewer in assessing our manuscript in detail and the useful recommendations listed. We hope now to have addressed these concerns in the revised manuscript.

**Reviewer #3:**

*This manuscript entitled “Impact of b-defensin 103 (DEFB103) copy number variation on bull sperm parameters and post-insemination uterine gene expression” and submitted by Kieran Meade investigate the possible role of DEFB103 as a potential marker of fertility in bovine. Firstly, the authors examine the DEFB103 copy number (CN) in spermatozoa of bulls classified as low or high fertility, followed by investigating various parameters of sperm derived from low fertile bulls with known CN values (low, intermediate, and high). Additionally, the authors examine the differential impacts on uterine tissue gene expressions 12h post-insemination.
Overall, the manuscript is well-written. It addresses a subject of interest to better contribute to selecting dairy cows. The authors provide sufficient background information to understand the study's rationale and cite the most appropriate references to support the study goal. The experimental procedures are well described, and relevant techniques are explained. However, the high CN diversity among high-fertility bulls appears to have excluded this group from the remaining study. I believe the complete omission of including high fertile bulls in the second part of the study may have removed the most crucial information related to the abundance of CN in low vs high fertile bulls. This leaves the reader asking how the results obtained with low fertile bulls in the second study will be significant. Regardless, the findings are appropriately discussed within the context of the current literature. In my opinion, the absence of high fertile bull, at least a comparison between low CN values, is a low point of the study, which will remain of interest to those in the closely related field of science.*

***Author response:*** We thank the author for their positive comments and for recognising the merit in this work. We were limited by the lack of genetic diversity in the high fertility bull group and unfortunately, we did not have enough biological replicates within each CN group to conduct further detailed analysis on high fertility bulls. We recognise this is a limitation of the study and have included a caveat in the discussion of the revised manuscript.

*Additional comments are listed below.
Specific comments
Table 1. Could the authors add new lines after H10 and L10, providing the average(±sd/sem) of all values listed above? This will allow for a fast grasp of the information related to comparing H vs L.*

***Author response:*** This detail has now been added as requested.

*Could the authors provide an image of the artificial mucus they made? This would be of interest to those desiring to replicate it.*

***Author response:*** Artificial mucus was made by diluting sodium hyaluronate solution (MAP-5, Lab-stock MicroServices, Ireland) with phosphate-buffered saline. We can provide an image of the mucus but it is clear and we don’t think it would add to the paper. This type of artificial mucus has been used across a large number of published papers studies to assess sperm-mucus penetration.

*A clarification is needed to prepare the explants: “These were cultured in M199: - what was the final volume of the co-incubated explants and sperm? – Why were the 75 ul taken for? Counting loose sperm or something else.*

***Author response:*** The explants were pipetted through 75 uL of M199 media to remove loosely bound sperm. We then only counted bound sperm on the explants. Please see revised methods for added clarity.

*Other clarification is needed for the heifer breed selection. Four breeds are mentioned, but only two were used per individual bull of low, intermediate, or high CN. Could this mean that each CN value group included all four heifer breeds, with two bred with the single bull? How do the authors end up with 18 heifers? - Would two heifers per bull be enough for complete reliance to generate reliable data?*

***Author response:*** Each CNV treatment was balanced according to heifer breed. We designed the study with the experimental unit being the heifer (i.e the effect of sperm on their uterus) and therefore we had an n of 6-8 heifers per CNV treatment. We believe having two heifers per bull enhanced the study and while we would have liked to have more heifers per bull these type of *in vivo* studies on cattle are expensive and always have logistical constraints.

*Overall, the absence of page and line numbers makes a thorough review with specific comments difficult.*

***Author response:*** We apologise for this oversight. Numbers are now included in the revised document.

**Reviewer #4:**

*The study explores the relationship between copy number variation (CNV) in the β-defensin 103 (DEFB103) gene and various aspects of bull fertility, including sperm motility, binding, and uterine gene expression post-insemination. Using a combination of in vitro and in vivo methodologies, the authors investigate molecular and physiological effects of DEFB103 CNV on fertility outcomes in Holstein-Friesian bulls. The findings suggest that lower DEFB103 CN is associated with improved sperm motility and enhanced uterine responses, providing a novel genetic marker for fertility assessment in cattle.
A. Abstract and Introduction
1. Abstract: The abstract is slightly dense. Emphasizing key findings would improve accessibility for a broader audience.*

***Author response:*** Considering the comments by another reviewer, we have now reworked the abstract. Perhaps the reviewer could check if this is sufficient.

*2. Introduction: The link between β-defensin functionality and fertility could be expanded with more emphasis on prior studies involving other β-defensin genes in cattle.*

***Author response:*** This detail has now been added as requested.

*B. Methods
1. Bull Selection: While the selection criteria for bulls are explained, providing more details about how "low" and "high" fertility bulls were identified from field fertility data would add clarity.*

***Author response:*** This detail has now been added as requested.

*2. Statistical Analysis: Explain the rationale for choosing an FDR threshold of 0.1 for differential gene expression.*

***Author response:*** This detail has now been added as requested.

*3. Sample Size Justification: The study relies on a small sample size (e.g., n=3 per CNV class for some analyses). A discussion of the limitations posed by this and how they were mitigated would be valuable.*

***Author response:*** This detail has now been added as requested.

*C. Results and Discussion
1. Figure 1 (binding to oviductal epithelium) could benefit from clearer labeling and legends explaining the significance of observed trends.*

***Author response:*** This detail has now been added as requested.

*2. While the discussion addresses findings in-depth, it occasionally becomes speculative (e.g., roles of CNN3 and RPS3A in embryo implantation). Stronger links to experimental data are needed to support such claims.*

***Author response:*** We have reduced the claims made in the results and discussion and hope this addresses the reviewer’s comments.

*3. Comparative discussion of findings with other β-defensin studies across species could highlight the broader relevance of the results.*

***Author response:*** This detail has now been added as requested.

*4. Terminology Consistency: Terms like "low fertility" and "low DEFB103 CN" need consistent usage throughout to avoid confusion.*

***Author response:*** This detail has now been added as requested.

*5. Data Accessibility: RNA-seq data availability is noted, but providing a direct repository link in the main text or supplementary information would be helpful for replication purposes.*

***Author response:*** A link is made available in the manuscript to the RNA-seq data.

*4. Broader Impacts: This study offers valuable insights for livestock genetics and fertility management. While the findings are preliminary, the implications for breeding strategies and identifying molecular markers for fertility are significant. Further research with larger sample sizes and across diverse breeds would strengthen these findings.*

***Author response:*** In light of the comments received, we have included a caveat in the discussion section of the revised manuscript to make this point. The final sentence reads “These findings illustrate the likely impact of *DEFB103* CNV on multiple aspects of bovine reproduction and suggests that further dissection of the fertility roles of β-defensins in a larger panel of cattle and breeds is now warranted”. We hope that these and all other modifications address the reviewers comments.
